# Clinical risk associated with COVID-19 among 86000 patients with congenital heart disease

**DOI:** 10.1136/openhrt-2023-002415

**Published:** 2023-12-13

**Authors:** Simon G. Williams, Simon Frain, Hui Guo, Matthew J Carr, Darren M Ashcroft, Bernard D Keavney

**Affiliations:** 1 Division of Cardiovascular Sciences, School of Medical Sciences, Faculty of Biology, Medicine and Health, Manchester Academic Health Science Centre, The University of Manchester, Manchester, UK; 2 Division of Population Health, Health Services Research and Primary Care, School of Health Sciences, Faculty of Biology, Medicine and Health, The University of Manchester, Manchester, UK; 3 Centre for Pharmacoepidemiology and Drug Safety and NIHR Greater Manchester Patient Safety Research Collaboration, The University of Manchester, Manchester, UK; 4 Manchester Heart Institute, Manchester University NHS Foundation Trust, Manchester, UK

**Keywords:** CONGENITAL HEART DISEASE, COVID-19, Electronic Health Records

## Abstract

**Objective:**

To determine the magnitude of any excess risk of mortality and hospitalisation due to COVID-19 infection in patients with congenital heart disease (CHD) in the UK healthcare system.

**Methods:**

Matched case–control study within the Clinical Practice Research Datalink study of anonymised general practice records in the National Health Service in England. Patients with CHD were stratified for disease severity according to the European Society of Cardiology guidelines. Presence of a positive COVID-19 test, hospitalisation with a diagnosis of COVID-19 and COVID-19-related mortality were compared in case and control groups.

**Results:**

86 441 patients with CHD and 335 839 controls were studied. Of patients with a positive COVID-19 test, patients with CHD were more likely than controls to be hospitalised (22.4% vs 14.5%; OR=1.77 (95% CI 1.60 to 1.96); p=2.11e−28) and suffer COVID-19-related death (6.1% vs 3.8%; OR=1.60 (95% CI 1.35 to 1.89); p=7.00e−08). The excess risk of COVID-19 hospitalisation and death rose with increasing physiological severity of CHD (presence of pulmonary vascular disease and/or cyanosis), rather than anatomical complexity.

**Conclusions:**

In this study of the COVID-19 pandemic experience, using population health records in over 86000 patients with CHD in England, patients with CHD with COVID-19 were at around 50–75% higher risk of hospitalisation and mortality compared with matched controls with COVID-19. We provide the first primary care-derived estimates for COVID-19 hospitalisation and case-fatality rates in patients with CHD. Some factors predictive of worse COVID-19 outcome in general populations (such as non-white ethnic group), and other CHD-specific comorbidities (such as pulmonary hypertension), influenced outcomes among patients with CHD.

**WHAT IS ALREADY KNOWN ON THIS TOPIC**

- Studies in the USA and in mainland Europe have estimated the excess risk from COVID-19 infection to hospitalised patients with congenital heart disease, and these estimates have varied widely. Large-scale data from the UK on this subject are thus far absent. The hospital focus of previous studies also precludes the estimation of hospitalisation and case-fatality rates in the general population of patients with CHD, and may have excluded some important events occurring in the community, such as sudden death.

**WHAT THIS STUDY ADDS**

- Using the Clinical Practice Research Datalink to study the general practice healthcare records of 86 441 CHD cases, we have quantified the excess risk of death and hospitalisation from COVID-19 among patients with CHD in the UK healthcare system. Further, we have shown that patients with more severe CHD diagnoses were more likely to suffer adverse events than were patients with milder CHD diagnoses; this relationship was substantially influenced by associated diagnoses of pulmonary vascular disease.

**HOW THIS STUDY MIGHT AFFECT RESEARCH, PRACTICE OR POLICY**

- We show that patients with CHD are at higher risk of hospitalisation and death in the context of COVID-19 infection. This study predated the onset of large-scale population vaccination in the UK; our study agrees with previous data suggesting it may be particularly important for patients with CHD to participate in vaccination programmes, including for any newly emerging variants. Our study also shows the need for further work to develop strategies to mitigate the excess mortality risk among patients with CHD admitted to hospital with COVID-19.

## Introduction

As of the end of 2022, there had been >175 000 COVID-19-related deaths in the UK with >20 million confirmed positive infections.^1^ Congenital heart disease (CHD) affects ~9 in 1 000 liveborn babies with an increasing global incidence.^2 3^ Several previous studies have examined the excess risk conveyed by a diagnosis of CHD in the setting of COVID-19 infection.^4–10^ These studies have predominantly been conducted in US-based cohorts and all have used an ascertainment approach limited to hospital treated patients. The previous studies have yielded highly disparate estimates of the risks of COVID-19-related death in hospitalised patients with CHD, ranging from 2.3%^4^ to 15%,^8^ among a total number of patients with CHD dying with COVID numbering about 750 across all studies. Absence of a matched control group in some of these studies also limits the possible inferences around the relative increase in COVID-19 risk to patients with CHD, compared with those who do not have CHD. No previous study has used community-derived data to investigate this question.

Here we investigate the clinical risk associated with COVID-19 infection in patients with CHD in the UK health system using the Clinical Practice Research Datalink (CPRD), containing primary care medical information from >20 million people in England. We identify 86 441 patients with CHD and, comparing with 335 839 matched controls, use information on positive PCR COVID-19 test surveillance, hospital episode data and death registration to examine the relationship between CHD complexity and COVID-19 infection severity in the contexts of age, sex, ethnicity and associated pulmonary vascular disease.

## Methods

### Data sources

**Figure 1:**
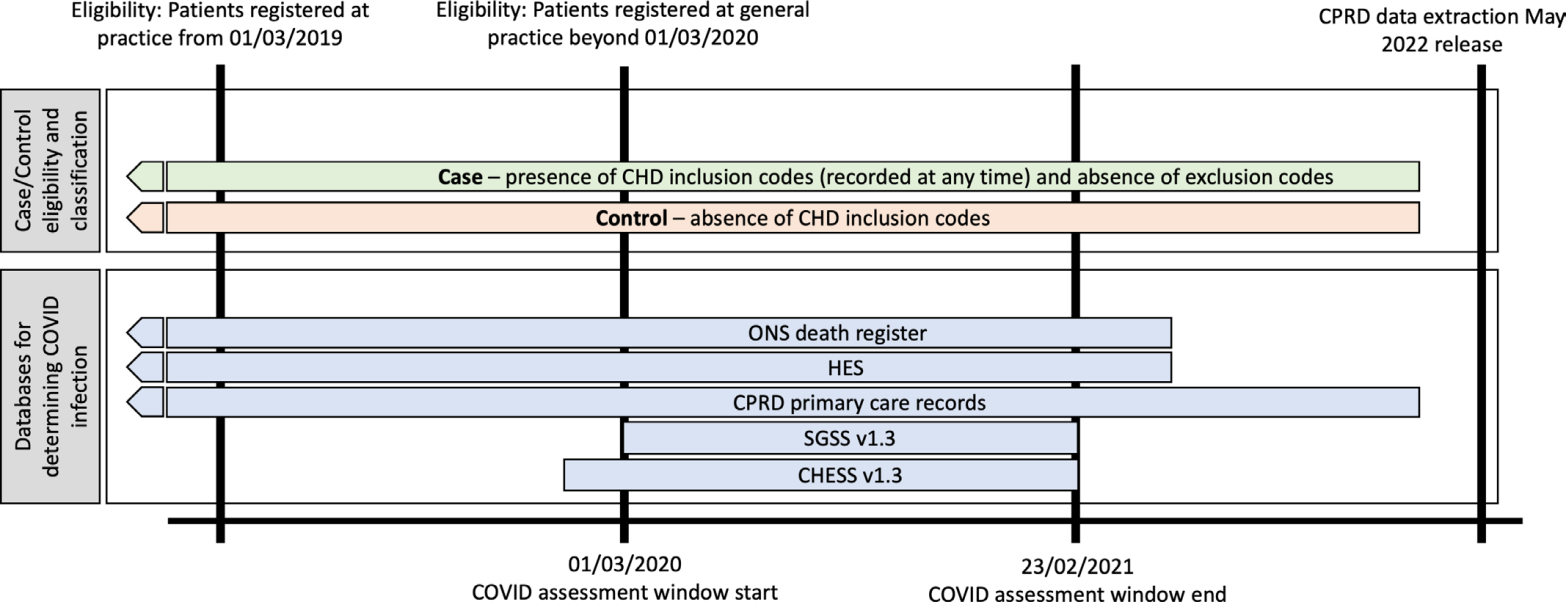
Schematic of timelines for determining study cohort and COVID-19 assessment window given data availability. CHD, congenital heart disease; CHESS, COVID-19 Hospitalisations in England Surveillance System; CPRD, Clinical Practice Research Datalink; ONS, Office of National Statistics; SGSS, SGSS, Second-Generation Surveillance System.

Primary care data were obtained from the CPRD Aurum database May 2022 release. Linkage of our study samples to additional databases was carried out by the CPRD using established multistep algorithms matching on features such as national health service (NHS) number, sex, date of birth and postcode. Linkages were obtained from hospital episode statistics—Admitted Patient Care (HES APC) v2.8 and, where applicable, Office of National Statistics (ONS) death registration data (v2.6—set January 2022). Additionally, specific COVID-19 positive virology data were obtained from the Second-Generation Surveillance System v1.3 (SGSS) database and the COVID-19 Hospitalisations in England Surveillance System v1.3 (CHESS). These linked datasets have varying coverage periods, defining our COVID-19 infection and hospitalisation assessment window. CHESS and SGSS cover 1 January 2020 and 1 March 2020, respectively, up to 23 February 2021. The ONS death registrations and hospital episode statistics (HES) data cover from 1997 and 1998, respectively, up to 31 March 2021. Additional COVID-19 positive test results from the CPRD Aurum dataset were, in turn, restricted to events occurring up to and including 23 February 2021 (figure 1).

### CHD case/control classification and clinical coding

We assembled matched case/control cohorts using primary care electronic health records from CPRD. General practices contributing data to CPRD with a ‘last collection date’ post 1 March 2020 were selected, resulting in patients from 1429 practices for analyses and representing ~19% of the UK population. A list of CHD-classifying SNOMED CT/EMIS codes can be found in online supplemental table 1. Any patient with at least one of these inclusion codes was initially selected. Prior diagnosis of a medical condition with the potential to confound or obscure a CHD diagnosis, for example, infection or inflammation of the heart muscle or connective tissue disorders which could affect heart valves, was identified and patients removed from further analysis (online supplemental table 2). Additionally, patients in whom valve disease could not be confidently identified as congenital in origin were removed from the potential CHD patient dataset. Chiefly, these patients had aortic valve (AV) disease first diagnosed over age 65 where bicuspid aortic valve (BAV) was not explicitly identified as the diagnosis. We considered AV disease first diagnosed before age 65 as BAV disease, an approach previously validated in the UK Biobank resource by ourselves and others.^11–13^ Candidate patients with CHD were further filtered to only include those identified as meeting CPRD-defined quality standards, whose registration at the general practice extended beyond 1 March 2020 and who had been registered at that practice for at least 1 year prior to 1 March 2020. Our final CHD case cohort consisted of 86 441 patients for analysis (online supplemental figure 1).

10.1136/openhrt-2023-002415.supp2 Supplementary data

10.1136/openhrt-2023-002415.supp1 Supplementary data

To select suitable control patients for this study, samples were matched to cases based on gender, nearest age (98.6% of controls were +/−2 years) and ethnicity. Patient ethnicity was simply assigned where no conflicting ethnicities were present in their records. Instances of conflicting ethnic assignment were resolved by selecting the dominant category (based on counts) and in the event of a tie, assigning the last recorded category of dominance. Importantly, potential controls were removed if they contained any diagnostic code from the CHD-defining list. To remove any potential regional bias, the pool of matched controls was filtered to include only CPRD patients registered at the same practice as the case to which they were matched, defined by being a patient of that practice for at least 1 year prior to 1 March 2020, and classed as eligible for linkage. As with case samples, they had to be from general practices with a last data collection date post 1 March 2020. In total, 335 839 unique controls were matched at an average of 3.9 control patients per case patient.

Individual CHD-defining codes were grouped into broader categories of severity according to the definitions adopted by the European Society of Cardiology (ESC), which focus on physiological consequences of CHD lesions.^2^ The assignment of ‘mild’, ‘moderate’ or ‘severe’ is shown in online supplemental table 1. According to the ESC guidelines, septal defects are found in both the mild and moderate categories but partitioned based primarily on their size, with larger unrepaired defects or those with additional abnormalities falling into moderate complexity. As septal defect size is difficult to resolve using the available diagnostic codes, we classed septal defects with evidence of surgical repair as being ‘moderate’, with unrepaired defects assigned to the ‘mild’ category in the absence of other associated abnormalities. When other associated abnormalities were present in an individual with an unrepaired defect, the patient was classed ‘moderate’.

Another aspect of complexity in the ESC classification is that the presence of pulmonary vascular disease or cyanotic CHD, regardless of the abnormality, results in a ‘severe’ classification. To determine these diagnoses in our cohort, we used the codes listed in online supplemental table 3.

### COVID-19 severity

Patients were classified as having a positive COVID-19 infection if they were present in the SGSS or CHESS databases. Additionally, the presence of ICD10 codes U07.1 or U07.2, indicating a positive COVID-19 test or clinical diagnosis of COVID-19, in the primary care, HES or death registration databases was used to identify patients with evidence of COVID-19 infection and, where appropriate, COVID-19 hospitalisation or death.

### Statistical analysis

**Figure 2:**
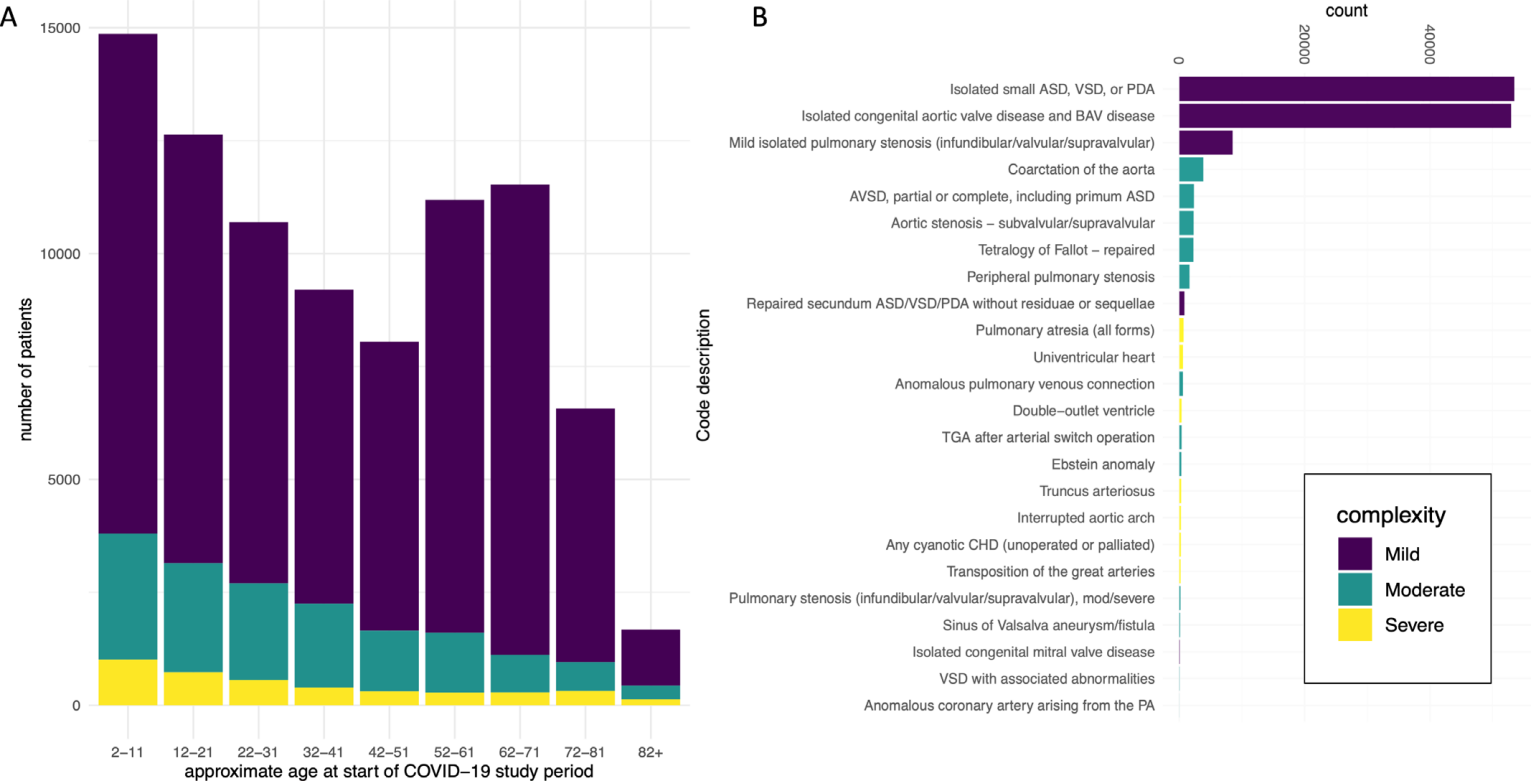
(A) CHD case cohort (N=86 896) split into age categories and displaying the number of mild, moderate and severe complexity patients with CHD in each. (B) A histogram showing the ESC guidelines, CHD complexity subgroups and their relative abundance among the CHD cohort. ASD, atrial septal defect; AVSD, atrioventricular septal defect; BAV, bicuspid aortic valve; CHD, congenital heart disease; ESC, European Society of Cardiology; PA, pulmonary atresia; PDA, patent ductus arteriosus; TGA, transposition of the great arteries; VSD, ventricular septal defect.

Outcomes measured between case and controls were: positive COVID-19 test results, hospitalisation due to COVID-19 and death due to COVID-19. Matched case and control samples were compared using conditional logistic regression (using R package ‘survival’) stratified by age, sex, ethnicity and general practice (as ‘pracid’ code). To determine the risk associated with age group, ethnicity and sex, each in turn was removed from the stratifying group and treated as a predictor. Where subgroups were assessed for differences, tests for interaction were performed to determine whether any difference was statistically significant. For the case-only analysis assessing the relative contribution of risk factors among patients with CHD, a logistic regression model was fit including binary covariates of age group (>50 years/2–50 years), sex (male/female), ethnicity (non-white/white/unknown), CHD complexity (severe/mild or moderate) and pulmonary hypertension/cyanosis diagnosis (presence/absence). As well being implicated in increased COVID-19 risk, patient age group (2–50 years and >50 years) is reflective of differences in the CHD cohort with an increase in patient numbers aged between 50 and 65, primarily due to increased diagnoses of BAV (see figure 2). The age group threshold was therefore set at this age. Samples with missing data in the covariates/stratifying variables were excluded from analysis.

This study adheres to the REporting of studies Conducted using Observational Routinely-collected Data (RECORD) statement^14^ (see Supplementary RECORD checklist).

## Results

### Demographics of study patients with CHD

A comparison of the demographics of the cohorts is shown in table 1, indicating well matched case and control samples. The age profile of 86 441 CHD cases identified for this study, subdivided by CHD severity, is shown in figure 2A. There is a progressive decrease in patient numbers from early childhood through to age 50, chiefly due to fewer moderate and severe CHD conditions in the cohort after age 11, which is in keeping with known life expectancy reduction of patients with complex CHDs, despite modern cardiological and surgical care.^15^ Patient numbers aged between 50 and 65 increase, primarily due to increased diagnoses of BAV, the most common CHD condition. During cohort selection, we excluded patients with AV disease presenting for the first time >65 without the explicit diagnosis of congenital BAV, as these were likely due to age-related degeneration of a trileaflet AV. Figure 1B shows the numbers of patients with CHD by diagnostic group. As anticipated, by far the two largest groups are that comprising small atrial septal defect, ventricular septal defect or patent ductus arteriosus; and BAV. Nevertheless, sizeable numbers of less common and more severe lesions are represented, for example, 2053 patients with tetralogy of Fallot and 3375 patients with aortic coarctation(online supplemental table 1). Overall, the cohort studied here is representative of the incidence of different CHD subtypes in England and other populations.

**Table 1:** A comparison of the demographics between case and control cohorts

	CHD cases	Controls
Total patients	86 441	335 839
Mean age (years)	40.6	40.7
Sex		
Female	41 384 (48.4%)	162 639 (48.4%)
Male	44 607 (51.6%)	173 200 (51.6%)
Ethnicity		
Asian	5674 (6.6%)	21 932 (6.5%)
Black	2661 (3.1%)	10 134 (3.0%)
Mixed	1142 (1.3%)	4116 (1.2%)
Other	575 (0.7%)	2029 (0.6%)
Unknown	13 439 (15.5%)	52 222 (15.5%)
White	62 950 (72.8%)	245 406 (73.1%)

### Patients with CHD have more severe COVID-19 infection outcomes

Patients with CHD were more likely to have a positive COVID-19 diagnosis than patients without CHD; 4.2% (N=3628) of patients with CHD tested positive for COVID-19 infection compared with 3.6% (N=12 243) of patients without CHD (OR=1.18 (1.14 to 1.21); p=1.06e−23). 22.4% of patients with CHD with a positive COVID-19 test were hospitalised (813/3628), compared with 14.5% (1776/12 243) of controls with a positive COVID-19 test (OR=1.77 (95% CI 1.60 to 1.96); p=2.11e−28). Among COVID positive cases and controls, 6.1% (N=222) of patients with CHD died compared with 3.8% (N=466) of controls (OR=1.60 (95% CI 1.35 to 1.89); p=7.00e−08). Of those admitted to hospital with COVID, death occurred (either in hospital or within 30 days of discharge) in 22.9% of patients with CHD (N=186) and 19.8% of controls (N=352) (OR=1.24 (1.01 to 1.52); p=4.32e−02).

### Heterogeneity in COVID-19 hospitalisation and death risk among patients with CHD

**Figure 3:**
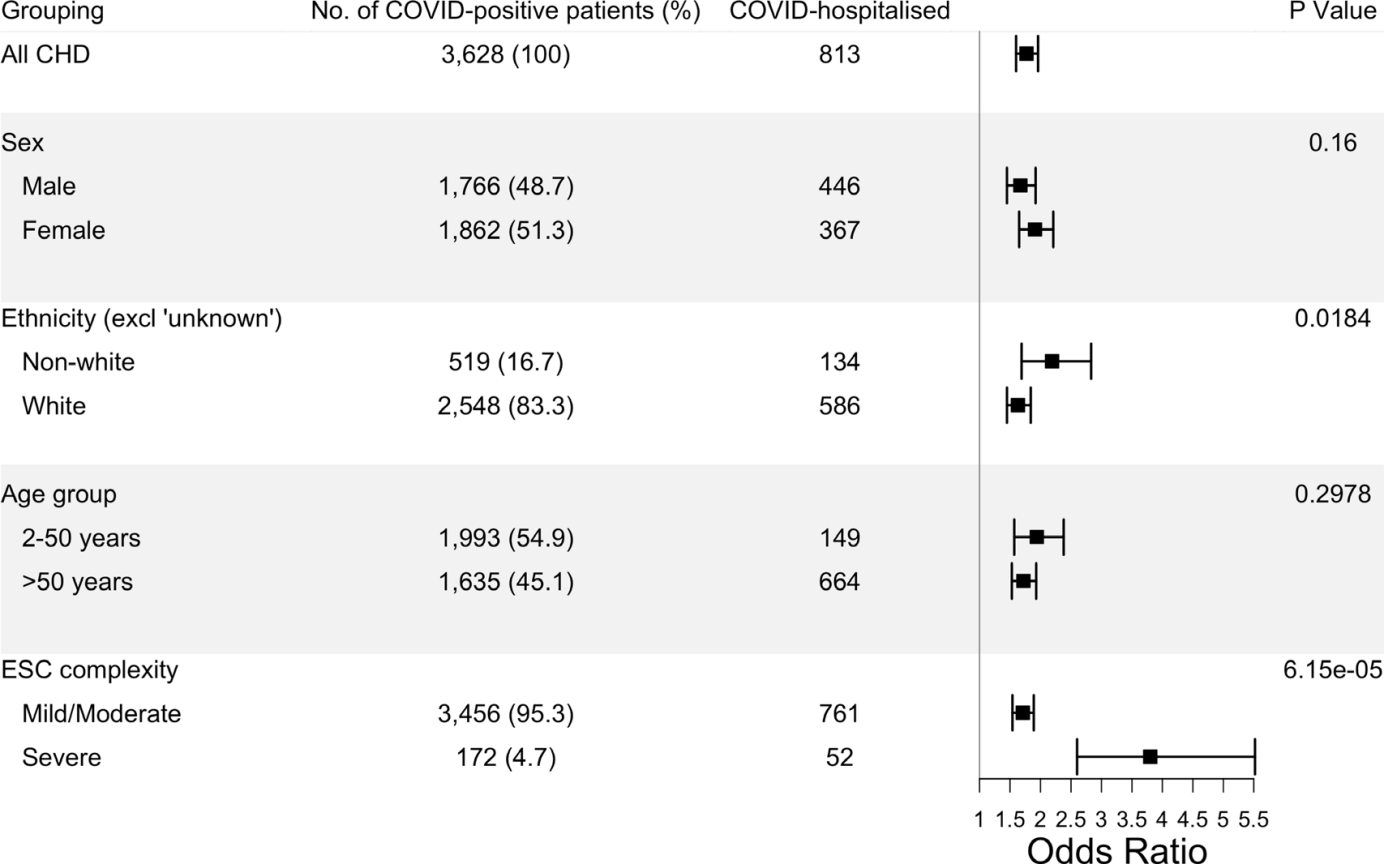
Exploration of heterogeneity in risk of hospitalisation between subgroups of patients with CHD and matched controls. P values are for tests of interaction. CHD, congenital heart disease.

We explored heterogeneity in risk of COVID hospitalisation and death among prespecified subgroups of patients with CHD and matched controls. There was no heterogeneity in risk of hospitalisation with respect to sex and age group (2–50 years and >50 years), whereas non-white ethnicity (including ‘Asian’, ‘black’, ‘mixed’ and ‘other’) and severe CHD complexity category were associated with higher hospitalisation risks (p=0.0184 and 6.15e−05, respectively; figure 3)

**Figure 4:**
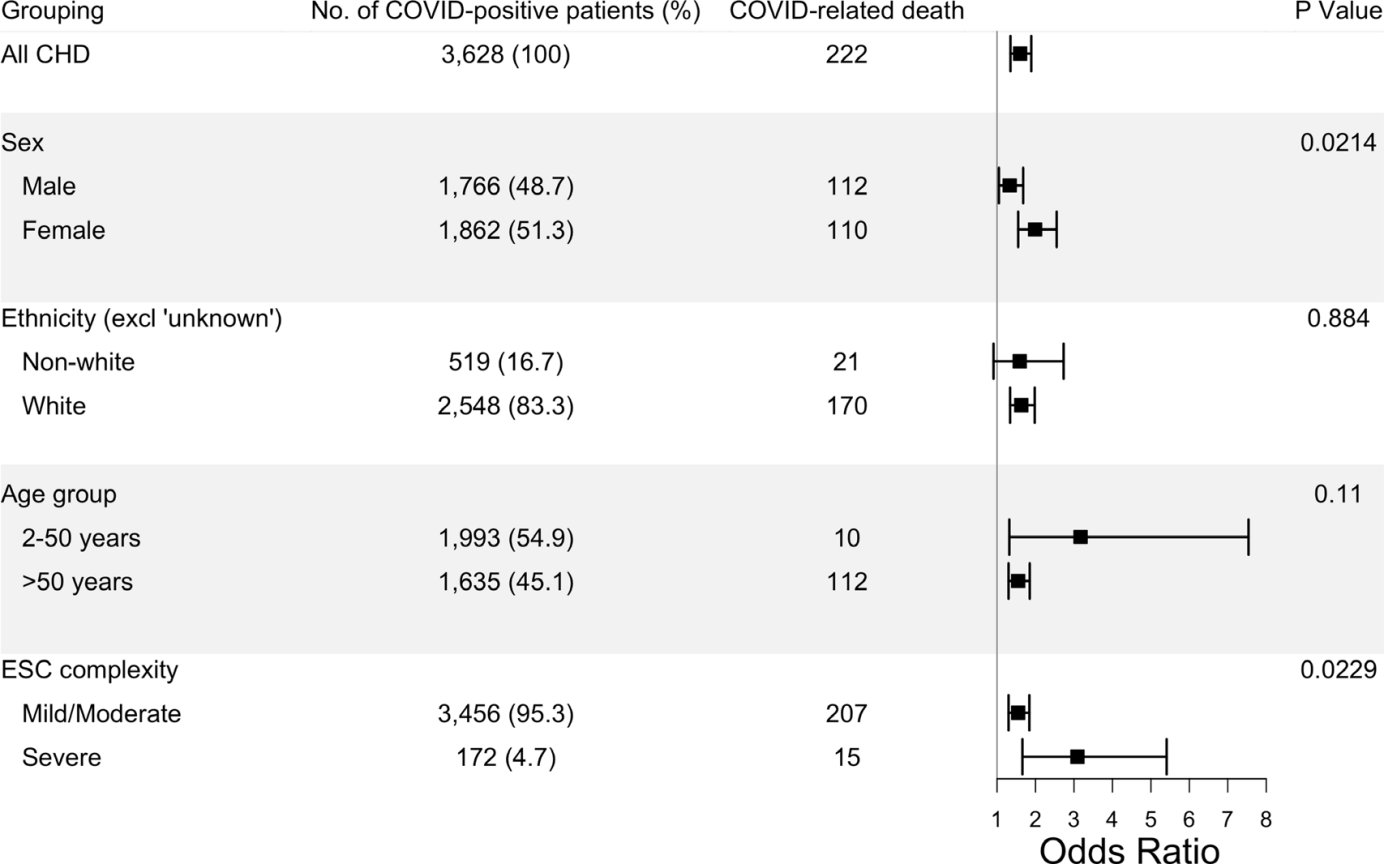
Exploration of heterogeneity in risk of death between subgroups of patients with CHD and matched controls. P values are for tests of interaction. CHD, congenital heart disease.

There was no significant heterogeneity in risk of COVID-19 death between ethnicities, and between age group categories (figure 4). However, the heterogeneity test for sex indicated that the increased risk among females with CHD compared with females without CHD exceeded that among male patients with CHD compared with males without CHD (OR=1.99 (1.55 to 2.55) for females; OR=1.33 (1.05 to 1.68) for males; heterogeneity p=0.0214). As with risk of hospitalisation, there was also a significant trend towards more severe ESC complexity being associated with increased risk of death (p=0.0229; mild/moderate OR=1.55 (1.30 to 1.84) and severe OR=3.09 (1.66 to 5.41)). The subgroup analyses of deaths are chiefly presented for the purpose of hypothesis generation, due to the small numbers in some subgroups.

### Pulmonary hypertension as a predictor of COVID-19 severity in patients with CHD

**Figure 5:**
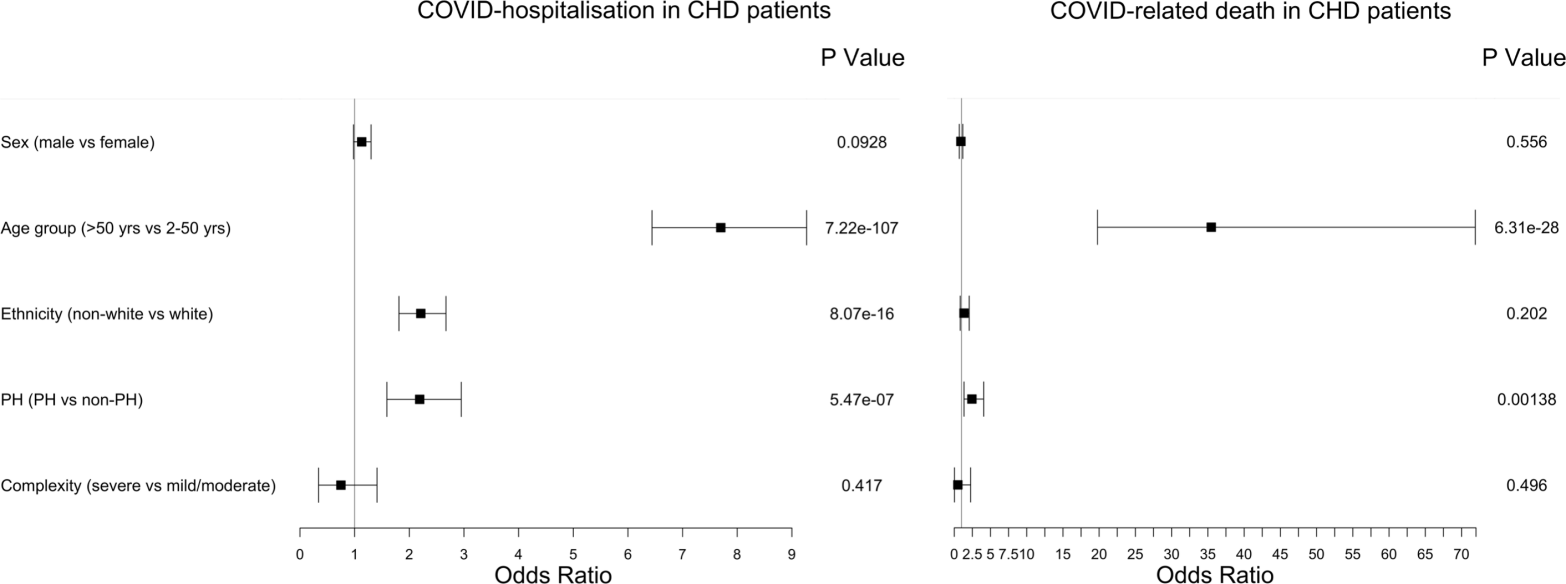
Among patients with CHD (case-only analysis), the risk of COVID-19 hospitalisation and death for a number of risk factors after adjustment in a combined model. In each case, the OR of the first factor is compared with the second used as reference. For example, for ‘sex’ the OR represents the relative increase or decrease in the odds for males compared with females.

The ESC complexity guidelines classify patients with CHD with pulmonary vascular disease or cyanotic CHD as severe, regardless of anatomical defect. Since previous studies had indicated the presence of pulmonary hypertension significantly modulated COVID-19 risk in CHD, we assessed this in our cohort. We extracted patients with CHD, with positive COVID-19 infections, without pulmonary hypertension/cyanosis (PH) (N=3525) and those with any CHD with PH (N=103). COVID-19 hospitalisation was more common in patients with CHD with PH compared with patients with CHD without PH (43.7% and 21.8%, respectively; p=2.83e−07). Likewise, COVID-19-related death occurred in 13.6% (N=14) patients with CHD with PH and COVID-19 compared with 5.9% (N=208) patients with CHD with COVID-19 infections but without PH (p=2.68e−03). The influence of several contributing factors was considered for hospitalisation and COVID-19-related death in the CHD patient group (figure 5). It should be noted these analyses are comparing risk within the CHD cohort only and are not matched case–control analyses; they facilitate comparison of our data with previous papers that have not used control groups.^4 7 9^ The largest risk factor for both hospitalisation and death was age, with the >50 years group showing significantly greater risk compared with younger patients with CHD (hospitalisation—OR=7.70 (6.44 to 9.27); p=7.22e−107 and death—OR=35.5 (19.77 to 7.19); p=6.31e−28). Ethnicity also showed a significant difference, with non-white patients with CHD at greater risk of hospitalisation (OR=2.21 (1.81 to 2.67); p=8.07e−16); there was a non-significant trend towards higher risk of death among non-white patients with CHD, in limited numbers. Comparing CHD patients with pulmonary vascular disease and/or cyanosis to those without, we find PH CHD patients at twofold to threefold greater risk of both hospitalisation and death. In contrast, the severe complexity CHD phenotypes, once PH and cyanosis diagnoses were excluded, showed no increased COVID risk for hospitalisation or death over mild or moderate complexities.

## Discussion

Using the CPRD to assess COVID-19 outcomes in patients with CHD in England, we have shown that patients with CHD are more likely to experience more severe COVID-19 outcomes, in terms of hospitalisation and death, than patients without CHD. Our cohort consists of predominantly adult patients with CHD with mild conditions and is to-date the only reported case/control study with population level ascertainment of CHD COVID-19 cases. Thus, uniquely among studies thus far, we have estimated population-based case-hospitalisation and case-fatality rates among patients with CHD, which were high, at 0.95% and 0.26% respectively. Among patients with CHD with evidence of COVID-19 infection, the hospitalisation and fatality rates were also high 22.4% and 6.1%, respectively. These estimates were both significantly higher than among controls matched for certain known risk factors for poor COVID-19 outcome including age, sex, ethnicity and, as a proxy for any regional variation, general practice. We showed significant interaction between ethnicity and poorer COVID-19 outcomes among patients with CHD; that is, the additional risk associated with non-white ethnicity among our patients with CHD was yet more substantial than the additional risk shown in many previous studies to be associated with non-white ethnicity in the non-CHD population. We also showed significant interaction between sex and poorer COVID-19 outcomes among patients with CHD: female patients with CHD were significantly more likely to die.

Previous large-scale studies of this question have differed in their cohort sizes, ascertainment strategies and assessment modalities. Broberg *et al*^4^ assessed 1044 patients with CHD admitted to cardiology centres across the USA and Europe (and incorporated data from a previous study conducted by Lewis *et al*^7^). In this cohort, which included 24 COVID-19-related deaths among patients with CHD, a case/fatality rate of 2.3% (95% CI 1.4% to 3.2%) among hospitalised patients with CHD, similar to the general population, was found. Schwerzmann *et al*^9^ studied 105 hospitalised patients from a multicentre European network, of whom 13 suffered significant complications and 5 died, finding that risk factors for poor outcome with COVID-19 in the general population also affected outcome in patients with CHD. Downing *et al*^5^ assessed 421 hospitalised US patients with CHD, along with controls, for COVID-19 outcomes, ascertaining from a US hospital payer database. This study reported a higher mortality rate (11%) among hospitalised patients with CHD, than previous studies. Strah *et al*^10^ undertook retrospective review of a US hospital performance improvement database. Among 549 adult and child patients with CHD, 47 died, yielding a death rate of 3.8% among child patients and 10.5% among adult patients. Among children, there was an excess in COVID-19 deaths among patients with CHD compared with controls without CHD. There was no excess death among adults with CHD, although the adult control group in this study was significantly older than the CHD case group. Hospital complications and costs were higher among the patients with CHD. More recently, Raj *et al*^8^ assessed a cohort of 4219 patients with adult CHD hospitalised with COVID-19 ascertained from the US National Inpatient Sample database, among which 639 died. Mortality of 15.1% among the admitted cases was higher than in controls. Among patients with CHD hospitalised with COVID-19, we find the highest yet reported mortality, 23% (187/813). Multiple factors, which our data do not encompass, are likely to account for this higher mortality in the UK healthcare system than in previous reports. We also found a higher rate of COVID-19 positive tests among patients with CHD but can only speculate as to the cause of this; it may be that the testing threshold for patients with CHD was lower due to anxieties about worse outcomes that predated published data availability.

Our results also highlighted the key relevance of pulmonary hypertension complicating CHD in determining adverse outcomes. When pulmonary hypertension was accounted for, anatomical complexity had no further significant association with poorer COVID-19 outcomes among patients with CHD. In this regard, our data agree with that of Broberg *et al*,^4^ who showed that physiological conditions associated with CHD have a greater impact on COVID-19 response than anatomical complexity of the defect.

The excess risk of poorer outcomes in non-white patients with CHD with COVID-19, and among female patients with CHD with COVID-19, compared with white and male patients warrants further investigation to find ways to mitigate these inequalities.

### Study limitations

Limitations of data availability in terms of COVID-19 infection, hospitalisation and death reporting time periods (not extending beyond the end of March 2021) mean that we were unable to determine the long-term effects of COVID-19 in patients with CHD and the potential clinical implications of long-COVID-19. Another area for further investigation is the impact of COVID-19 vaccination on the CHD cohort. During the time window for this study, there were few patients who had received a dose of COVID-19 vaccination (88 case and 232 control patients) and therefore it was not possible to determine its effect on hospitalisation and COVID-19-related death. Future work using updated clinical databases will allow determination of the impact of vaccination and further inform health policy regarding the clinical management of patients with CHD in the context of COVID-19.

## Data Availability

Data may be obtained from a third party and are not publicly available. Primary care data were obtained from the Clinical Practice Research Datalink (CPRD) Aurum database May 2022 release, through application 20_000161. Access to the data is available once a project has been reviewed and approved by CPRD’s research data governance process. CPRD has ethics approval from the Health Research Authority (21/EM/0265) to support research using anonymised patient data.
