# Supplementary figures and images for: Clinical risk associated with COVID-19 among 86000 patients with congenital heart disease
Source: Open Heart. 2023 Dec 13;10(2):e002415. doi: 10.1136/openhrt-2023-002415 (PMC10729200; doi:10.1136/openhrt-2023-002415)

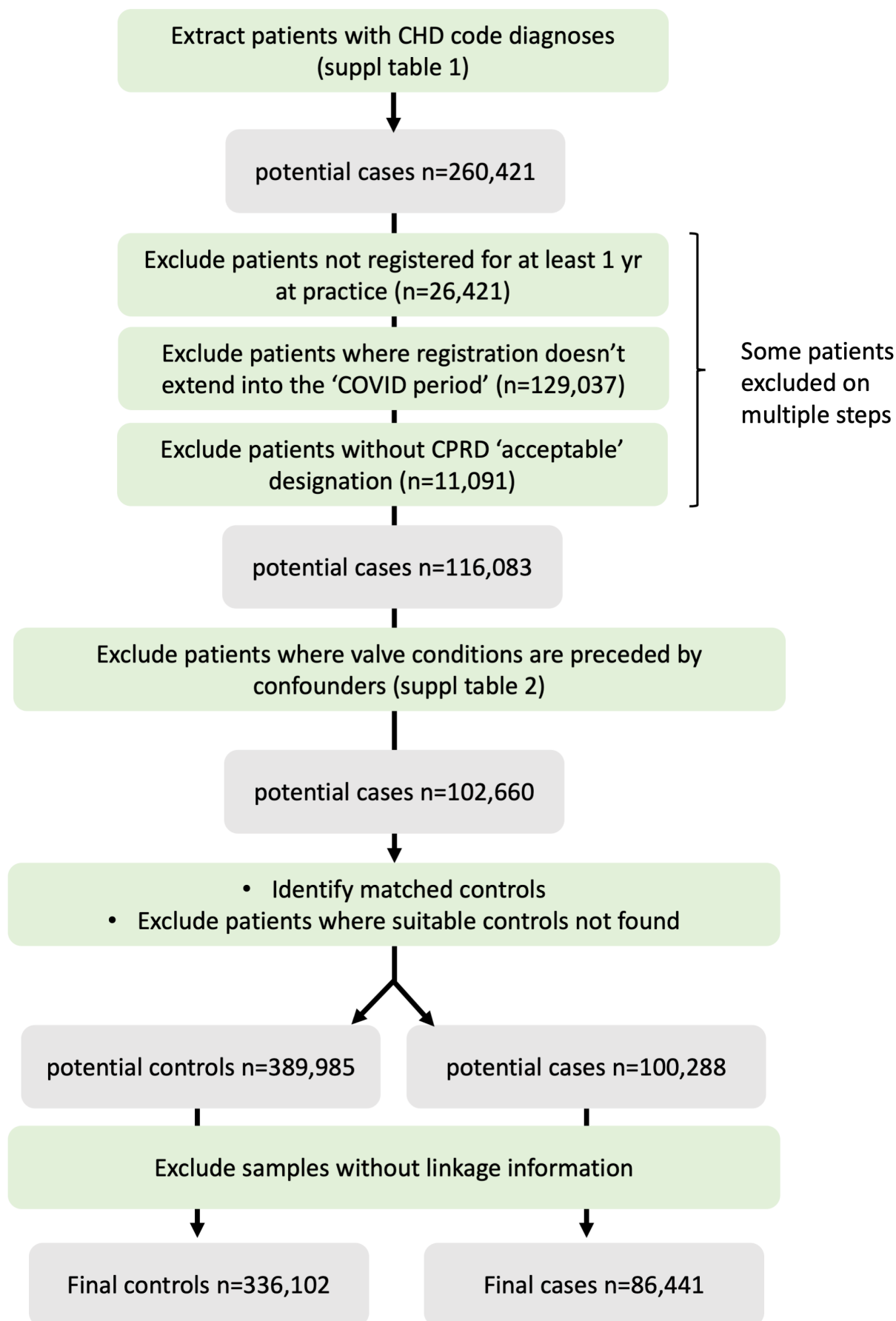

Supplement: Supplementary data [file openhrt-2023-002415supp001.pdf]
